# Commentary: Mobile and Interactive Media Use by Young Children: The Good, the Bad, and the Unknown

**DOI:** 10.3389/fpsyg.2017.00461

**Published:** 2017-03-28

**Authors:** Cedric Galetzka

**Affiliations:** Division of Cognitive Sciences, Psychology Department, University of PotsdamPotsdam, Germany

**Keywords:** embodied cognition, children's development, touchscreen devices, learning, smartphones

There seems to be a broad consensus among today's parents that early exposure to digital media is less enriching than real-life experiences (Wooldridge, 2016). While this concern may rightly apply to traditional media such as television, new interactive devices (e.g., smartphones and tablets), on the contrary, are often marketed as supplemental learning tools for children (Kirkorian et al., 2009; Christakis, 2014; Apple, 2016). However, Radesky et al. (2015) recently pointed out that research on the impact of interactive devices on children's cognition cannot keep up with the pace of technological advances. The most recent guidelines on recommended screen time were updated before the first tablets even made their way onto the market (Christakis, 2014). Supplementing Radesky et al. (2015), this commentary aims to clarify the influence of modern touchscreen devices on children's cognitive development from the perspective of embodied cognition.

Embodied cognition highlights that the development of cognitive processes crucially depends on active interactions between one's body and the environment (Barsalou, 1999; Thelen et al., 2001). These sensorimotor interactions are thought to form the basis for many high-level processes such as object recognition and decision-making (Smith, 2005; Rivière and David, 2013). Importantly, children in early developmental stages seem to build up fewer associations from interactions when merely observing an action being executed instead of performing it (Smith, 2005). Modern interactive devices address this concern in that they allow for active bodily interactions via touchscreens (Black et al., 2012; Christakis, 2014).

Radesky et al. (2015) note that research has been sparse on whether children can actually benefit from touchscreen use. However, recent studies indicate that children can transfer acquired knowledge from touchscreen interfaces to physical objects. Wang et al. (2016) compared the effectiveness of teaching children clock reading using an iPad touchscreen app, a toy clock, or a paper drawing. The researchers found that children learned equally well from interactive media and traditional toys, with both conditions outperforming the paper drawing group. Importantly, children were able to transfer learned skills from touchscreens to physical objects. The same has been found in a study teaching children how to solve the Tower of Hanoi problem (Huber et al., 2016). In sum, the typical transfer deficit observed with traditional media seems to be absent when children are actively engaged with devices via touchscreens (Strasburger, 2015; Huber et al., 2016).

However, going beyond Radesky et al. (2015), simple swiping and tapping motions on touchscreens seem impoverished compared to the complex hand movements that facilitate exploration of objects (Lederman and Klatzky, 1987; Spitzer, 2013). Confirming this suspicion, Crescenzi et al. (2014) compared children's performance on a drawing task in an iPad vs. paper condition. The researchers point out that touchscreens limit the amount of fingers available for object manipulation. Moreover, qualitative aspects of touch are reduced and important haptic information, such as surface texture, are completely absent. However, the authors note that iPads allow for new types of touch, including more complex touch sequences. Interestingly, a recent study found that touchscreen use in early childhood is correlated with fine motor control (Bedford et al., 2016). Nevertheless, the authors argue that negative effects on more complex motor processes might only become apparent in later stages of development. Therefore, developers of children's applications need to take principles of embodiment, such as allowing for more complex bodily interactions, into account in order to guarantee for a healthy cognitive development (Antle, 2009).

Supporting Radesky et al. (2015), while children are engaged with interactive media, they miss out on other potentially more fruitful activities that foster an understanding around them. However, the limited types of touch of today's smartphones could provide certain beneficial learning effects by replacing traditional forms of media, such as television (Christakis, 2014). Kirkorian et al. (2016) compared the performance of children aged between 24 and 36 months on a word learning task. The children were either instructed to watch a video from a touchscreen or use touch-based gestures during video presentation. The authors observed that especially younger children benefited from contingent touchscreen interactions that accompanied task-relevant information. Interestingly, this condition appeared to be counterproductive for the oldest children who participated in the experiment. In a follow-up study, Choi and Kirkorian (2016) argued that contingent touch-based interactions mainly facilitate learning in younger children by supporting selective attention mechanism. Future research needs to specify under which conditions contingent touchscreen responses supplement learning.

Moreover, Eisen and Lillard (2016) observed that children consistently prefer real-world objects, such as books, for learning over touchscreen devices. Importantly, children seem to grasp the interactional nature of touchscreens compared to traditional media but fail to conceive them as useful learning tools. The authors reasoned that children discount the learning value of interactive devices due to the circumstances they encounter them. Correspondingly, Radesky et al. (2014) already pointed out that parents mostly let their children use interactive media during routine tasks. Children would benefit from a more systematic approach to learning from touchscreens that takes these aspects into account. Recently, Kucirkova (2016) proposed a framework to bring developers, practitioners, and researchers together to design empirically based applications.

To sum up, the extent to which the advantages of real-life learning might be substitutable by touchscreen devices seems to be heavily context-dependent. Even in the absence of joint engagement, which was shown to greatly benefit learning according to Radesky et al. (2015), interactive media could represent useful supplementary learning tools in educational contexts (Kwok et al., 2016). In that sense, other fruitful approaches could also be to compare the effectiveness of touchscreen devices with other embodied learning approaches such as using active role-play to facilitate reading comprehension (Glenberg et al., 2004; Black et al., 2012). As with any other newly popularized technology, the true potential of smartphones is likely to be discovered along the way (Lovato and Waxman, 2016). Ultimately, insights from embodied cognition contribute to understanding how the touch in touchscreens supports the process of learning.

## Author contributions

The author confirms being the sole contributor of this work and approved it for publication.

### Conflict of interest statement

The author declares that the research was conducted in the absence of any commercial or financial relationships that could be construed as a potential conflict of interest.
